# Supplementary material for: Southern African Origin of HTLV-1 in Romania
Source: PLoS Negl Trop Dis. 2024 Aug 6;18(8):e0012337. doi: 10.1371/journal.pntd.0012337 (PMC11302904; doi:10.1371/journal.pntd.0012337)
Supplement: Attachment — Submitted filename: Reviewer_comments.docx [file pntd.0012337.s001.docx]

**PNTD-D-24-00050**

Comments to the editor and authors:

The article by Cassar et al is of great interest since it analyzes the molecular epidemiology of HTLV-1 in Romania, the only European country where HTLV-1 is endemic. However, several aspects must be reviewed before publication.

In more detail,

1. The work analyzes a very small number of patients and the conclusions derived can be confusing. It would be appreciated if the introduction expanded on information related to the epidemiology of HTLV in Romania, including information related to demographic aspects such as age, sex, origin, etc.
2. The discussion is very brief and provides little epidemiological explanation for the findings observed.
3. In the article, 3 identical sequences are mentioned (ROU8, ROU2, ROU5), which are also the same as the Romanian reference sequence RK12. Evidence of identical sequences not epidemiologically related suggests a founder effect. In addition, 2 other identical sequences are observed in the phylogenetic tree, ROU1 and ROU_PH630.
4. Case ROU2 also presented HBV co-infection. Some articles have suggested that the infection of children in Romania with a variety of blood-borne viruses during Ceausescu's communist dictatorship could explain this unexpected finding. The frequent use of non-sterile syringes and the lack of detection of blood agents in hematology products played an important role. (De Void D, Piñeiro-Carrero V, Goodman Z, Latimer J. Chronic active hepatitis B infection in Romanian adoptees. J Ped Gastroenterol Nutr 1994; 19: 431-6).
5. Case ROU7, whose mother is also HTLV positive, appears in the phylogenetic tree together with MN532074_Nig21. Information regarding the origin of the mother could help discuss this finding.
6. In order to explain the identity between sequences, some epidemiological data could be useful (ethnicity, place of residence in childhood, etc.)
