# Supplementary material for: Southern African Origin of HTLV-1 in Romania
Source: PLoS Negl Trop Dis. 2024 Aug 6;18(8):e0012337. doi: 10.1371/journal.pntd.0012337 (PMC11302904; doi:10.1371/journal.pntd.0012337)
Supplement: Attachment — Submitted filename: Responses to reviewers_22-05-24.docx [file pntd.0012337.s002.docx]

Dear reviewers,

Please find below the responses to your comments and suggestions which have been carefully considered. The necessary corrections have been made accordingly, as evidenced by the changes highlighted in the manuscript, and we hope that these changes address all the concerns you raised.

We sincerely thank you for your valuable comments, which reinforce the quality of our manuscript.

Yours sincerely,

Dr. O. Cassar and Pr. A. Gessain

**Methods**

**Reviewer #2**: The main method of this study consists in the molecular analysis of these new HTLV sequences. Therefore, it is essential to include in methods how the sequences were obtained, and all phylogenetic analysis done. This information is currently spread in results session. Authors need to include it in methods.

We have now included in the "Material and Methods" section two subsections. The first entitled “*HTLV-1 amplification and sequencing*” explains how the sequences were obtained (pages 7-8, lines 97-128) and the second called “*Phylogenetic analyses*”, details the phylogenetic analyses carried out (pages 8-9, lines 129-136).

    First statement of results belongs to methods. And authors need to expand briefly on the method used (reference 12).   “DNA samples were obtained from peripheral blood buffy coats. The LTR sequence was obtained for the 11 samples, as previously described (12)”

The first part of the results has been moved, as suggested by the reviewer, to the “Material and Methods” section. The methodology used to obtain complete genome sequences is now detailed on pages 7-8, lines 98-128.

    Figure 1 and 2 legends also contain important information that needs to be in methods.

The methodological information on phylogenetic analyses previously included in the legends to Figures 1 and 2 has been moved to the “*Phylogenetic analyses*” sub-section of the “*Material and* *Methods*” section on pages 8-9, lines 129-136.

    The following statement in results also should be in methods.

    “The DNA samples were subjected to four series of PCR (F1-F4) using both a high-fidelity hot-start DNA polymerase and primers designed from highly conserved regions for the major HTLV-1 subtypes. In brief, we obtained four different HTLV-1 proviral genomic regions, each about 2.5 kb in length: F1, LTR-Gag; F2, pro-pol; F3, pol-env and F4, tax-LTR. PCR products were then directly sequenced on both strands using a set of 16 specific primers (12). After alignment of sense and antisense sequences using the Clustal W algorithm (Mac Vector 18.6.1 software, Oxford molecular), comparison of each generated segment was implemented to derive a consensus sequence.”

As suggested by the reviewer, the sentences that appeared in the results section are now found in the “*Material and Methods*” section and the “*HTLV-1 amplification and sequencing* ” sub-section on pages 7-8, lines 98-128.

    Minor points:

    -	How many patients were tested for HBV?

Only five individuals were tested for HBV infection (ROU1, ROU2, ROU3, ROU7 and ROU9). This information is now specified in the text on page 7, lines 94.

    -	How many mothers were tested?

Only the mothers of patients ROU7 and PH630 could be tested and were found both HTLV-1 seropositive. This information is given in table 1 on page 11.

    -	Include in methods information about transfusion (the first time this is mentioned is in discussion). Was it done in Romania?

Information on transfusion events is now described in the “*Material and* *Methods*” section and the “*HIV and HBV screening and blood transfusion history*” sub-section. The location of these transfusions has also been specified (page 7, lines 92-96). Only two patients (ROU1 and ROU8) reported transfusion events performed in Romania during infancy.

    -	If available, it would be interesting to know how long they have been out of Romania.

We understand the interest in this question, but data on the time spent by these people outside Romania is not available.

**Reviewer #3**: There appear no problems in the objectives, methods and interpretation of the results.

    Ethical or regulatory requirements are properly met.

**Results**

**Reviewer #2**: Results

    Authors says that all sequences except ROU7 belong to a-TC-Mozambique – Southern Africa A subgroup. What about the outlier (ROU7)?

While all the sequences characterized belong to the a-TC-Mozambique - Southern Africa A subgroup, only the ROU7 sequence does not. This sequence is part of the HTLV-1a Transcontinental group but does not belong to any subgroup. This has now been specified in the manuscript on page 12, line 208.

    Authors did whole genome sequence of 10/11 sequences, please inform the reader which sample was missing.

The proviral genome of the strain corresponding to patient ROU8 could not be fully sequenced due to the small amount of DNA available. This is now specified on page 13, line 219.

    Authors say that stop mutations are quite frequent in ATL. This needs to go to discussion, and a reference is needed.

Following the reviewer's remark, we double-checked all the sequences we had characterized. During concatenation of the fragments of strain ROU8, one base was duplicated in an erroneous manner, resulting in a shift in the reading frame and the appearance of a Stop codon. Following this check, the supernumerary base was removed, and the Stop codon no longer exists. Consequently, the reference made in the text to the presence of stop codon in an ATL patient has been removed.

    Authors say that 9/10 Romanian strain form a specific “Romania” clade. Please add in the text which sample is the exception.

The sample not belonging to the "Romania" clade is ROU7, which is now specified on page 13, lines 231-232.

    The last paragraph of result can be moved to discussion.

As suggested by the reviewer, the last paragraph of the "results" section has now been moved to the "*Discussion*" section on page 14, lines 250-251.

    Suggest replacing “They” at the beginning of the second paragraph of results for something more specific Newly characterised sequences…

As proposed by the reviewer, we have reworded the sentence and replaced “They” by “The new characterized sequences….” On page 12, line 209.

**Reviewer #3**: Figure 1 and 2: The figures are blurred and the texts in the figures unreadable.

We apologize for the inconvenience. At the time of submission, the figures were clear and the text readable. However, during the creation of the PDF file by the software of the online submission site including both our manuscript and the figures in tiff format, the latter may have been modified and become blurred and illegible. At the time of this submission, we will check that the illustrations are legible. Furthermore, and in the upper corner of each right-hand submitted illustration you'll find a link to access each figure in readable pdf format.

    The analysis itself is appropriate and the results are described clearly.

**Conclusions**

**Reviewer #2**: Authors need to include a conclusion session.

Following the advice of the reviewer we have developed our discussion into a genuine conclusion that provides a clear and concise synthesis of the results as well as the implications and contributions of this study, while acknowledging its limitations and suggesting avenues for future research.

**Reviewer #3**: 1. Page 14: I understand that all the viruses identified in Romania seem to originate from Mozambique, but there is little discussion on the background and reasons for this. Is this because of immigrants? Also, given that multiple individuals have been infected, shouldn't there be a discussion of how and when the virus entered the country and how it spread within the country?

We understand the important questions raised by the reviewer, but we would like to make it clear that this molecular epidemiology and phylogeny work, by generating and comparing viral genomic sequences, was an attempt to understand the geographical origin of the HTLV-1 viral strains present in patients living in Romania. This is not a clinical and descriptive epidemiological study aimed at investigating the routes of introduction of this virus into Romania, nor its modes of dissemination in this country. Answers to these questions will require specific studies by our Romanian colleagues at a later date.

2. Page 14 and figure 2: The possible origin of the remaining one specimen should be discussed

As stated in the response to the previous question, it is challenging for us to speculate on the origin of the ROU7 sample in the absence of precise epidemiological data concerning this patient and her relatives (her mother in particular).

3. Page 14, lines 11-13: Since many cases of ATL from horizontal infection have been confirmed in Japan, should the wording be weakened?

As suggested by the reviewer, the sentence regarding the development of ATL only after virus acquisition during childhood has been softened to specify that there is also a possible occurrence of ATL following horizontal virus acquisition in adults and young adults but in a smaller proportion. A reference by Watanabe T supporting this observation has been added on page 14, lines 256-257 and quoted in the references (ref#17 on page 17, lines 329-330).

**Editorial and Data Presentation**

**Reviewer #1:** (No Response)

**Reviewer #2:** (No Response)

**Reviewer #3**: As mentioned above, the figures in the PDF are very poor to understand what are described. This may be due to the problems in making PDF, however, the clear figures need to be provided to the reviewers.

We apologize for the inconvenience caused by the poor quality of the figures, which may have hindered their readability and interpretation. This is indeed due to the formatting of the article by the online journal's website, which generated the final PDF file.

**Summary and General Comments**

**Reviewer #2**: This manuscript brings novel and interesting data that sheds light on HTLV-1 spread to Romania, a high endemic country in Europe. The molecular analysis is done at high standard and shows convincing evidence of a recent African origin.

However, some methods are currently described in result session or Figure legends. Discussion can also be expanded.

We are grateful to the reviewer for acknowledging that our results provide new and interesting data regarding HTLV-1 in Romania. We have considered all the comments in order to thoroughly restructure the manuscript so that the methods and results sections are now better separated, and the conclusion/discussion is more detailed.

Authors could include discussion about current situation of HTLV response in Romania. Are they testing Blood donors? Are they testing organ donors? They can also discuss strategies that can limit the spread of this virus in Romania (as they suggested this should be done).

Another interesting point to discuss is how to limit spread to other countries. Recent blood donor data indicates high prevalence of HTLV in Moldova and brings concern about other countries within the region where no HTLV prevalence data is available. Authors have extensive experience on HTLV epidemiology and could expand on this topic. Another question that this study brings is if the origin of HTLV in Moldova is the same as they have revealed for Romania.

We thank the reviewer for his/her comment and remarks. However, as previously mentioned, this molecular epidemiology and phylogeny study aimed to understand the geographical source of HTLV-1 viral strains found in patients residing in Romania through the generation and comparison of viral genomic sequences.

It is important to note that this is not a clinical or descriptive epidemiological investigation targeting the pathways of HTLV-1 introduction into Romania or its dissemination within the country. Addressing these inquiries will necessitate dedicated studies by our Romanian colleagues in the future.

To our knowledge, blood donor screening has been mandatory in Romania since 1999 and his information has been specified on page 6, line 74.

**Reviewer #3**: It is significant that the origin of HTLV-1 in Romania has been clarified.

However, there appears some room for improvement in interpretation and discussion of the data、in addition to the poor quality of the figures.

We thank the reviewer for his/her comment and hope that we have done our best in the revised version of the manuscript we are submitting to make the interpretation and discussion of the results we have obtained even clearer and more understandable.

We will do our best to improve the quality of the illustrations for this submission. Unfortunately, this depends above all on the software that generates the overall submission file in PDF format. However, it is possible to access a readable version of each illustration by clicking in the top right-hand corner of the image.

**Answers to the reviewer's questions attached to the Editor email.**

The article by Cassar et al is of great interest since it analyzes the molecular epidemiology of HTLV-1 in Romania, the only European country where HTLV-1 is endemic. However, several aspects must be reviewed before publication.

In more detail,

1. The work analyzes a very small number of patients and the conclusions derived can be confusing. It would be appreciated if the introduction expanded on information related to the epidemiology of HTLV in Romania, including information related to demographic aspects such as age, sex, origin, etc.

We thank the reviewer for his/her suggestion. We have provided some information that better details the available information concerning the epidemiology of HTLV-1 and associated diseases (on page 6, lines 72-77). However, this study is not a clinical and descriptive epidemiological aimed at investigating the routes of introduction of this virus into Romania, nor its modes of dissemination in this country. It is a molecular epidemiology and phylogeny study, generating and comparing viral genomic sequences, to understand the geographical origin of HTLV-1 viral strains present in patients living in Romania.

The answers to these pertinent questions will require specific studies by our Romanian colleagues.

2. The discussion is very brief and provides little epidemiological explanation for the findings observed.

The discussion has been more detailed, to further explain the results, we have obtained. However, we also pointed out that based on the data available at present, we cannot demonstrate, or even suggest, how this virus entered Romania and then spread there. We have underlined that there is, however, a certain similarity between the situation regarding the spread of HTLV-1 and that which existed a few decades ago in Romania for the epidemics of HIV and hepatitis B, particularly among children (pages 14-15, lines 262-271).

3. In the article, 3 identical sequences are mentioned (ROU8, ROU2, ROU5), which are also the same as the Romanian reference sequence RK12. Evidence of identical sequences not epidemiologically related suggests a founder effect. In addition, 2 other identical sequences are observed in the phylogenetic tree, ROU1 and ROU_PH630.

The results of phylogenetic studies must be interpreted with care. Although strains ROU1 and PH630 are identical, they are only identical on a 485-bp portion of the LTR. Analysis based on concatenation of the *gag-pro-pol-env* genes shows that the two strains are close, but quite distinct. Only strains ROU2 and ROU5 are identical in the 485-bp LTR portion and on the 6,366-bp portion analyzed (the DNA of strain ROU8 being in insufficient quantity to obtain the complete sequence).

4. Case ROU2 also presented HBV co-infection. Some articles have suggested that the infection of children in Romania with a variety of blood-borne viruses during Ceausescu's communist dictatorship could explain this unexpected finding. The frequent use of non-sterile syringes and the lack of detection of blood agents in hematology products played an important role. (De Void D, Piñeiro-Carrero V, Goodman Z, Latimer J. Chronic active hepatitis B infection in Romanian adoptees. J Ped Gastroenterol Nutr 1994; 19: 431-6).

We thank the reviewer for his/her comment. Indeed, it is tempting to think that there is a certain similarity between the situation concerning the spread of HTLV-1 and that which existed a few decades ago in Romania for the epidemics of HIV and hepatitis B, particularly among children. This possibility was suggested in the conclusion (Page 14, lines 262-265) and two articles by Haukenes, G *et al*. and Hersh, BG *et al*., have been quoted on page 15, line 265.

5. Case ROU7, whose mother is also HTLV positive, appears in the phylogenetic tree together with MN532074_Nig21. Information regarding the origin of the mother could help discuss this finding.

It would have been interesting to have epidemiological data on the relatives of infected patients, and in particular on the origin of the mother of patient ROU7, who was infected with HTLV-1. Unfortunately, this information is not available.

6. In order to explain the identity between sequences, some epidemiological data could be useful (ethnicity, place of residence in childhood, etc.).

As the reviewer suggests, data concerning certain epidemiological data such as ethnicity, place of residence in childhood, etc. could have been useful. Unfortunately, we do not have this type of information at our disposal, which is why we suggested that only solid, in depth epidemiological studies specifically seeking for risk factors for HTLV-1 acquisition in the Romanian population and the families of infected individuals, combined with historical data, will be needed to try to better understand the origin of the current situation (page 15, lines 266-269).
